# Synergistic antibacterial effects of closantel and its enantiomers in combination with colistin against multidrug resistant gram-negative bacteria

**DOI:** 10.3389/fmicb.2024.1374910

**Published:** 2024-05-02

**Authors:** Tongyan Ding, Zeyu Guo, Liangxing Fang, Wenying Guo, Yuxi Yang, Yafei Li, Xiarong Li, Limin He

**Affiliations:** ^1^National Risk Assessment Laboratory for Antimicrobial Resistance of Animal Original Bacteria, South China Agricultural University, Guangzhou, China; ^2^Guangdong Laboratory for Lingnan Modern Agriculture, Guangzhou, China; ^3^Inspection and Testing Center for Domestic Animal Products (Guangzhou), Ministry of Agriculture and Rural Affairs, Guangzhou, China; ^4^Guangdong Provincial Key Laboratory of Veterinary Pharmaceutics Development and Safety Evaluation, College of Veterinary Medicine, South China Agricultural University, Guangzhou, China; ^5^Institute of Quality Standard and Monitoring Technology for Agro-products of Guangdong Academy of Agricultural Sciences, Guangzhou, China

**Keywords:** closantel, stereoselectivity, colistin, reverse drug resistance, gram-negative bacteria

## Abstract

Drug combinations and repurposing have recently provided promising alternatives to cope with the increasingly severe issue of antibiotic resistance and depletion of natural drug molecular repertoires that undermine traditional antibacterial strategies. Closantel, an effective adjuvant, reverses antibiotic resistance in gram-negative bacteria. Herein, the combined antibacterial enantioselectivity of closantel is presented through separate enantiomer studies. Despite yielding unexpected differences, two closantel enantiomers (*R*, *S*) increased colistin activity against gram-negative bacteria both *in vitro* and *in vivo*. The fractional inhibitory concentration indices of *R*-closantel and *S*-closantel combined with colistin against *Pseudomonas aeruginosa*, *Klebsiella pneumoniae*, and *Escherichia coli* ranged from 0.0087 to 0.5004 and from 0.0117 to 0.5312, respectively. This difference was further demonstrated using growth inhibition assays and time-killing curves. Mechanistically, a higher intracellular concentration of *R*-CLO is more effective in enhancing the antimicrobial activity of combination. A mouse cutaneous infection model confirmed the synergistic stereoselectivity of closantel. This discovery provides novel insights for developing precision medication and containment of increasing antibiotic resistance.

## 1 Introduction

The “One Health” approach, aiming to achieve the overall health of humans, animals, and the environment, has become a focal point for most international organizations and countries worldwide (Gao, 2021; Gruetzmacher et al., 2021). Antimicrobial drugs and drug resistance have become the focus of attention as an important part of One Health (Bazzi et al., 2022; Ramsamy et al., 2022). In recent years, the problem of antimicrobial resistance has become a serious issue, especially on members of the microbiota of humans and domestic animals, but also in environments heavily polluted with antibiotics (Liu et al., 2019, 2021; Sulis et al., 2021). The global death toll from multidrug-resistant (MDR) bacteria and “superbugs” mediated by mobile genetic elements, such as plasmids and transposons, is expected to reach 10 million by 2050 (Ma et al., 2021; Kaiser et al., 2022). This is especially true with the emergence of the plasmid-mediated mobile colistin (CST) resistance gene (*mcr-1*) (Liu et al., 2016), in 2015, which encodes phosphoethanolamine transferase, the *mcr-1* gene has spread to more than 40 countries and regions through environment, vector animal, animal production. MDR-gram-negative bacteria (GNB) are increasingly becoming resistant to the last line of defense, i.e., polymyxins (Kai and Wang, 2020; Zhu et al., 2021). Conjugative plasmids can self-transfer between cells, leading to the horizontal transmission of bacterial resistance to polymyxins between different species, further exacerbating the global resistance crisis (Liu et al., 2016; Feng, 2018). Therefore, the control of MDR GNB to prevent the further generation of resistance is very necessary.

However, the development of antibacterial drugs is far slower than the speed at which bacteria acquire resistance. Therefore, as new antibacterial drugs cannot be obtained in a short period, there is an urgent need to explore new strategies to overcome MDR. Studies in recent decades have shown that some FDA-approved non-antibiotic drugs exert significant synergistic antibacterial effects with antibiotics, highlighting the repurposing of old drugs and drug combinations as a feasible way to combat MDR. Recent reports have indicated that anthelmintic salicylanilides, such as niclosamide and closantel (CLO), can eradicate gram-positive bacteria, including methicillin-resistant *Staphylococcus aureus* (minimum inhibitory concentration, MIC <0.5 μg/mL) and *Enterococcus faecalis* (MIC <2 μg/mL) (Pauk et al., 2013; Rajamuthiah et al., 2015).

CLO, an anthelmintic drug approved for veterinary use, has been shown to enhance the bacterial killing effectiveness of polymyxin B against MDR *A. baumannii*, *P. aeruginosa*, *K. pneumoniae*, and *E. coli* (Domalaon et al., 2019). It highlights the significance of CLO in controlling the global spread of plasmid-mediated CST resistance. More recent studies have identified CLO as a novel allosteric inhibitor of human Taspase1 (Luciano et al., 2021). All these reports show the importance of reuse old drugs under the circumstances that the development of new drugs is becoming more and more difficult. However, its toxic effect has been a hot spot that attracts much concern. The stereoselectivity of drug enantiomers greatly influences its pharmacology, efficacy and toxicity (Benez et al., 2013; Zhang et al., 2020). Thus, understanding the stereoselectivity of enantiomers is crucial for future assessments of environmental contamination, food safety, and global health.

For instance, Elder et al. (2022) investigated the stereoselectivity of bacterial resistance to chloramphenicol acetyltransferase and determined the role of stereochemistry in the environmental fate of chloramphenicol. Castringnano et al. studied the enantiomeric composition of ofloxacin in aquatic environments, revealing enrichment of *S*-(−)-ofloxacin in the receiving waters of a wastewater treatment plant (WWTP), indicating potential stereoselectivity degradation of ofloxacin by bacteria within the WWTP (Liu et al., 2012). Xue et al. reported that the xenobiotic pollutant epoxiconazole altered the structure and metabolism of soil microorganisms with significant stereoselectivity, primarily driven by 2*R*, 3*S*-(+)-cis-epoxiconazole (Xue et al., 2022).

**Figure 1:**
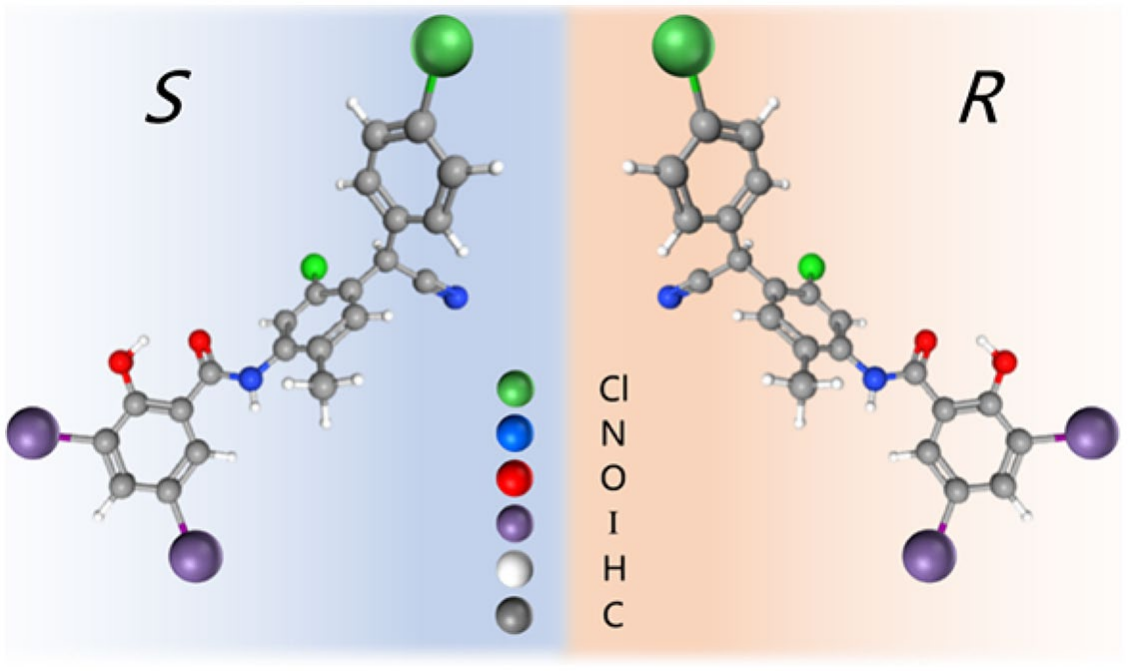
Molecular structure of closantel enantiomers.

Based on the above-mentioned research on chiral drugs and previous studies on CLO enantiomers in our laboratory (Saleh et al., 2021), the *in vitro* antibacterial activity of CLO, *R*-CLO, and *S*-CLO either alone or in combination with CST was systematically evaluated by combined antibacterial, growth inhibition assay, and time-killing curves against both CST-susceptible and insensitive GNB in the present study. On one hand, the current study helps us better understand the mechanism by which CLO works; on the other hand, this difference in stereoselectivity of CLO helps us better develop new drugs and reduce toxic effect, thereby provide more possibilities for its clinic use (Figure 1).

## 2 Experimental section

### 2.1 Materials

Standard CLO (purity, >98.1%) was purchased from Dr. Ehrenstorfer GmbH Company (Augsburg, Germany). *R*-CLO and *S*-CLO (purity, >98%) were provided by Guangzhou Yan Chuang Biotechnology Development Co. Ltd (Guangzhou, China). Standard CST (purity, >99%) was purchased from Beijing Puboxin Biotechnology Co. Ltd (Beijing, China).

### 2.2 Bacterial strains and growth conditions

Reference CST-susceptible *E. coli* ATCC 25922 strains and five clinical CST-resistant strains (*E. coli* 44, *E. coli* 55, *P. aeruginosa* PA02, *P. aeruginosa* PA05, and *K. pneumoniae* K83) were maintained in our laboratory. Two CST-resistant *E. coli* strains used in this study were confirmed to harbor the plasmid-mediated CST-resistance gene *mcr-1*. These organisms were cultured in Luria–Bertani broth (Boucher et al., 2009) at 37°C with shaking at 180 rpm.

### 2.3 *In vitro* pharmacodynamic studies

#### 2.3.1 *In vitro* susceptibility testing

*In vitro* antibacterial activity of the drugs was assessed using the broth microdilution susceptibility test according to the Clinical and Laboratory Standard Institute guidelines. The MICs for all the clinical isolates were determined using the broth microdilution assay in 96-well plates with Mueller–Hinton broth (MHB). All tests were performed at least in triplicate. Bacterial cultures grown overnight were then diluted in saline to achieve a 0.5 McFarland turbidity. The diluted bacterial culture was further diluted at 1:100 in MHB for inoculation. The drugs were twofold serially diluted in MHB and incubated with equal volumes of the bacterial inoculum at 37°C for 18 h. Wells with or without bacterial cells were used as positive or negative controls, respectively. *E. coli* ATCC 25922 was used as the control strain.

#### 2.3.2 Checkerboard assay

Experiments were performed on 96-well plates. CST was twofold serially diluted as shown on the y-axis, while CLO, *R*-CLO, or *S*-CLO was twofold serially diluted as shown on the x-axis, to create a matrix where each well consisted of a combination of both antimicrobial agents at different concentrations. Bacterial cultures were diluted in MHB and inoculated in each well to achieve a final concentration of approximately 1 × 10^5^ CFU/mL. Wells containing MHB with or without bacterial cells were used as positive and negative controls, respectively. Bacterial growth was examined by visual inspection after 18 h of incubation.

FIC of CST was calculated by dividing the MIC of CST in the presence of CLO by the MIC of CST alone. Similarly, FIC of CLO was calculated by dividing the MIC of CLO in the presence of antibiotic by the MIC of CLO alone. The FICI was the summation of both FIC values. A FICI of ≤0.5, synergy; 0.5–1, additivity; 1.0–4.0, indifferent (non-interactive); > 4, antagonistic.

#### 2.3.3 Growth inhibition of bacterial suspensions

Growth inhibition assays of GNB strains with a starting inoculum of 1 × 10^5^ CFU/mL were conducted on MHB in the presence of 2 μg/mL CLO and 0.1 μg/mL CST (sub-MIC) alone or in combination. The cultures were incubated at 37°C with shaking at 180 rpm for 24 h, and the bacterial growth was determined by measuring the OD every hour during the first 12 h, and then at 14, 16, 18, and 24 h at 600 nm.

#### 2.3.4 Time-killing curves of bacteria

Killing kinetics were studied in tubes containing MHB with bacteria (≈ 1 × 10^6^ CFU/mL) and a single or combination drug. Final bacterial suspensions were cultured for 24 h at 37°C. Culture aliquots of 100 μL were removed at 1, 2, 3, 4, 8, and 24 h, serially diluted in 0.9% sterile saline solution and plated on agar for counting colonies following 18 h of incubation at 37°C. All experiments were performed with three biological replicates. The results of the CFU count are presented as mean ± SD of three samples.

### 2.4 Exploration of mechanism

#### 2.4.1 Scanning electron microscopy analysis

Take the bacteria in logarithmic growth phase (*E. coli* 44) and dilute to 1 × 10^6^ CFU/mL with MHB. Then they were treated with 2 μg/mL CLO, 2 μg/mL *R*-CLO, 2 μg/mL *S*-CLO, 0.1 μg/mL CST and their combinations at 37°C for half an hour. The MHB with no drugs served as a control. The above bacterial samples were fixed with 2.5% glutaraldehyde for 1 h at 4°C, washed three times with deionized water, and then resuspended in water to make a bacterial dispersion. The dispersions were transferred to the copper tape surface using a 0.5 mm capillary and air dried. In order to increase the conductivity, the surface of the sample was plated with gold by magnetron sputtering (20 mA, 30s). Microscopy was taken on an Apero 2 S HiVac. The mode of the secondary electron image is SE, the high voltage is 10 kV, the current is 0.2 nA, and the magnification is 30,000 times.

#### 2.4.2 Reactive oxygen species detection

To investigate the impact of CLO/CST on bacterial reactive oxygen species (ROS) production, we employed a fluorescent probe, DCFH-DA, to quantify ROS levels in *E. coli*. The ROS Assay Kit obtained from Beyotime Biotechnology facilitated this analysis. In a succinct procedure, bacterial solutions of *E. coli* 44 was separately incubated with 10 μM DCFH-DA at 37°C for 30 min and subjected to two washes with PBS buffer. Subsequently, various tubes received either a single drug or a combination of CST (0.1 μg/mL) with CLO (2 μg/mL), *R*-CLO (2 μg/mL), or *S*-CLO (2 μg/mL). Fluorescence intensity was measured after a 30 min incubation period, with excitation and emission wavelengths set at 488 nm and 525 nm, respectively.

#### 2.4.3 Detection of drug concentration in bacteria

The combined growth inhibition assay of 0.5 μg/mL CLO and 0.1 μg/mL CST on *E. coli* 44 was conducted, employing a starting inoculum of 1 × 10^5^ CFU/mL in MHB. The bacterial culture underwent incubation for 10 min and 2 h at 37°C with continuous shaking at 180 rpm. Subsequently, 0.5 mL of the incubated bacterial sample was transferred into a 1.5 mL Eppendorf tube, followed by centrifugation at 10,000 × g for 10 min at 4°C, with subsequent removal of the supernatant. The bacterial pellet underwent two washes with a 0.9% saline solution, after which the tube was placed in a − 80°C refrigerator for a 1 h bacterial freeze. To facilitate the transfer of bacterial cells, a micro-tube cutter was employed to trim the tube approximately 3 mm from the bottom, causing the section containing the bacterial cells to fall into a new eppendorf tube. Subsequently, 0.5 mL of acetonitrile was added to the tube, followed by vortexing for 3 min. The mixture was centrifuged at 10,000 × g for 10 min at 4°C. Post-centrifugation, 0.4 mL of the supernatant was pipetted and diluted once with the initial mobile phase. The resulting sample solution underwent filtration using a 0.22 μm organic filter membrane for subsequent LC–MS/MS analysis.

### 2.5 *In vivo* pharmacodynamic study

Animal experiments were conducted in accordance with the Canadian Council on Animal Care guidelines and approved by the South China Agricultural University Laboratory Animal Center (approval number: 2021c056). Seven 7-week-old female Kunming mice weighing 25 ± 3 g used in this experiment were housed in a sterile environment. Mice were obtained from the Hunan Slack Jingda Experimental Animal Co., Ltd. Isoflurane (1 to 3%) and CO_2_ were used to anesthetize and euthanize mice, respectively. Then, 10 mg/kg b.w. CLO, *R*-CLO, *S*-CLO, and 5 mg/kg b.w. CST was dissolved in 0.5% DMSO for skin toxicity testing before subcutaneous injection. Prior to injection, bacterial cells were washed twice with sterile saline solution (0.9% NaCl) and resuspended to 1 × 10^7^ CFU/mL. The bacterial suspension (50 μL) was injected under a shaved area of mouse skin. A group receiving bacterial challenge only served as a positive control, and the other groups received a single antibiotic (CST, CLO, *R*-CLO, or *S*-CLO) or a CST combination (CST combined with CLO, *R*-CLO, or *S*-CLO) treatment 1 h after the bacterial challenge by injecting different concentrations of CLO (50 μL). The abscess was monitored daily, and the length (L) and width (W) of the lesion were measured in each mouse three days after bacterial injection to calculate the abscess volume (V) using the following formula: $V=\left(\pi /6\right)\times L\times W^{2}$ . The skin abscess (including accumulated pus) was removed three days after bacterial infection and homogenized in 1 mL of sterile normal saline for 5 min. Then the bacterial counts were determined by serial dilution. Experiments were performed independently using six animals per group.

### 2.6 Statistics

All data from at least three biological replicates are shown as mean ± SD. One-way ANOVA was used to calculate *p*-values (**p < 0.01, ***p < 0.001).

## 3 Results

### 3.1 *In vitro* pharmacodynamic studies

#### 3.1.1 Closantel enantiomers selectively synergized with colistin against GNB

The antibacterial activities of CST, CLO, and its two enantiomers were evaluated, as well as the potential synergism of CLO and the enantiomers with CST against three kinds of GNB (Table 1). The MICs for CST against CST-resistant *E. coli*, *P. aeruginosa*, and *K. pneumoniae* strains ranged from 2 to 128 μg/mL, while that against the susceptible reference strain *E. coli* ATCC 25922 was 0.5 μg/mL. The MICs for CLO, *R*-CLO, and *S*-CLO against the above-mentioned GNB were higher than 256 μg/mL. When CLO, *R*-CLO, or *S*-CLO was combined with CST, the MICs for the mixture decreased below the breakpoint of sensitivity to CST-resistant GNB.

**Table 1:** FIC index of the combinations of CLO, *R*-CLO, and *S*-CLO with CST.

Strain	CLO	MIC_CLO_ (MIC_combo_), μg/mL	MIC_CST_ (MIC_combo_), μg/mL	FIC index	Interpretation
*P. aeruginosa* PA02	CLO	>256 (0.5)	128 (1)	0.0097	Synergy
	*R*-CLO	>256 (0.25)	128 (1)	0.0087	Synergy
	*S*-CLO	>256 (1)	128 (1)	0.0117	Synergy
*P. aeruginosa* PA05	CLO	>256 (0.25)	2 (1)	0.5009	Additivity
	*R*-CLO	>256 (0.125)	2 (1)	0.5004	Additivity
	*S*-CLO	>256 (0.5)	2 (1)	0.5019	Additivity
*K. pneumoniae* K83	CLO	>256 (0.25)	4 (1)	0.2509	Synergy
	*R*-CLO	>256 (0.125)	4 (1)	0.2504	Synergy
	*S*-CLO	> 256 (1)	4 (1)	0.2539	Synergy
*E. coli* 44 (*mcr-1*)	CLO	> 256 (4)	4 (0.5)	0.1406	Synergy
	*R*-CLO	>256 (2)	4 (0.5)	0.1328	Synergy
	*S*-CLO	>256 (8)	4 (0.5)	0.1562	Synergy
*E. coli* 55 (*mcr-1*)	CLO	>256 (8)	2 (0.5)	0.2812	Synergy
	*R*-CLO	>256 (4)	2 (0.5)	0.2656	Synergy
	*S-CLO*	>256 (8)	2 (1)	0.5312	Additivity
*E. coli* ATCC 25922	CLO	>256 (0.25)	0.5 (0.25)	0.5009	Additivity
	*R*-CLO	>256 (0.125)	0.5 (0.25)	0.5004	Additivity
	*S-CLO*	>256 (0.5)	0.5 (0.25)	0.5019	Additivity

CLO, closantel; R-CLO, R-closantel; S-CLO, S-closantel; CST, colistin.

The fractional inhibitory concentration indices (FICIs) ranged from 0.0087 to 0.5312 for the combination of CST with CLO or the enantiomers for the tested GNB. Three representative strains were selected from different genera and the standard strain *E. coli* ATCC 25922. For *P. aeruginosa* PA02, the fold change in the MIC of CST was up to 128-fold in the presence of 0.25 μg/mL *R*-CLO, whereas the concentrations of CLO and *S*-CLO required 2-fold and 4-fold *R*-CLO to achieve the same effect, respectively. Accordingly, the combination of *R*-CLO and CST had the strongest synergy (FICI = 0.0087), followed by CLO and CST (FICI = 0.0097) and *S*-CLO and CST (FICI = 0.0117).

**Figure 2:**
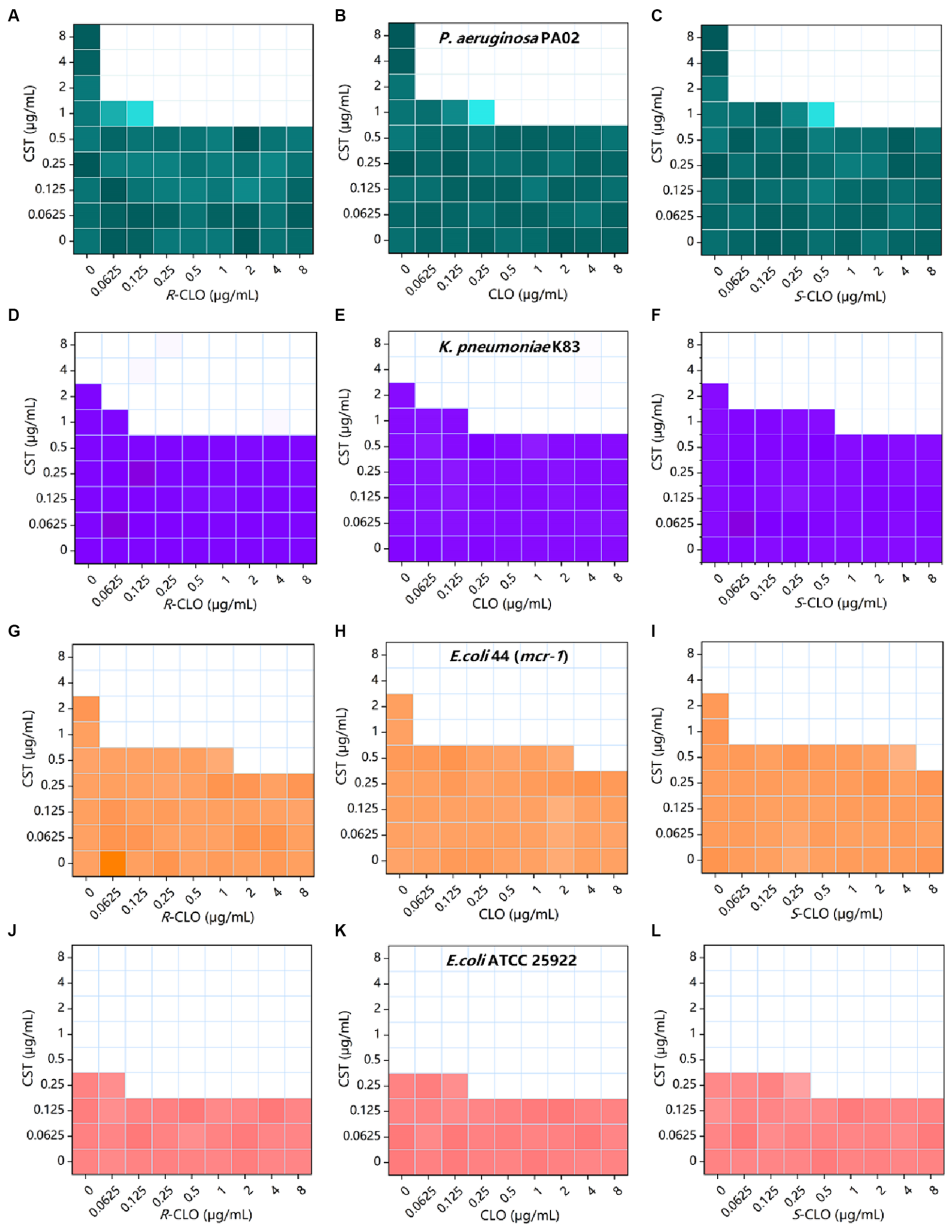
Combined inhibitory effect of 0 to 8 μg/mL CST and 0 to 8 μg/mL CLO, *R*-CLO, or *S*-CLO was tested against four different GNB strains in a checkerboard format. Bacterial growth is shown as a heat map (see Table 1 for further details). **(A–C)** Checkerboard analysis for CST combined with CLO and its two enantiomers against *P. aeruginosa* PA02. **(D–F)** Checkerboard analysis for *K. pneumoniae* K83. **(G–I)** Checkerboard analysis for *E. coli* 44 (*mcr-1*). **(J–L)** Checkerboard analysis for *E. coli* ATCC 25922. CLO, closantel; *R*-CLO, *R*-closantel; *S*-CLO, *S*-closantel; CST, colistin.

The bacterial growth heat map corresponding to the information in Table 1 is shown in Figure 2.

#### 3.1.2 Potent inhibition of drug combinations against bacteria

**Figure 3:**
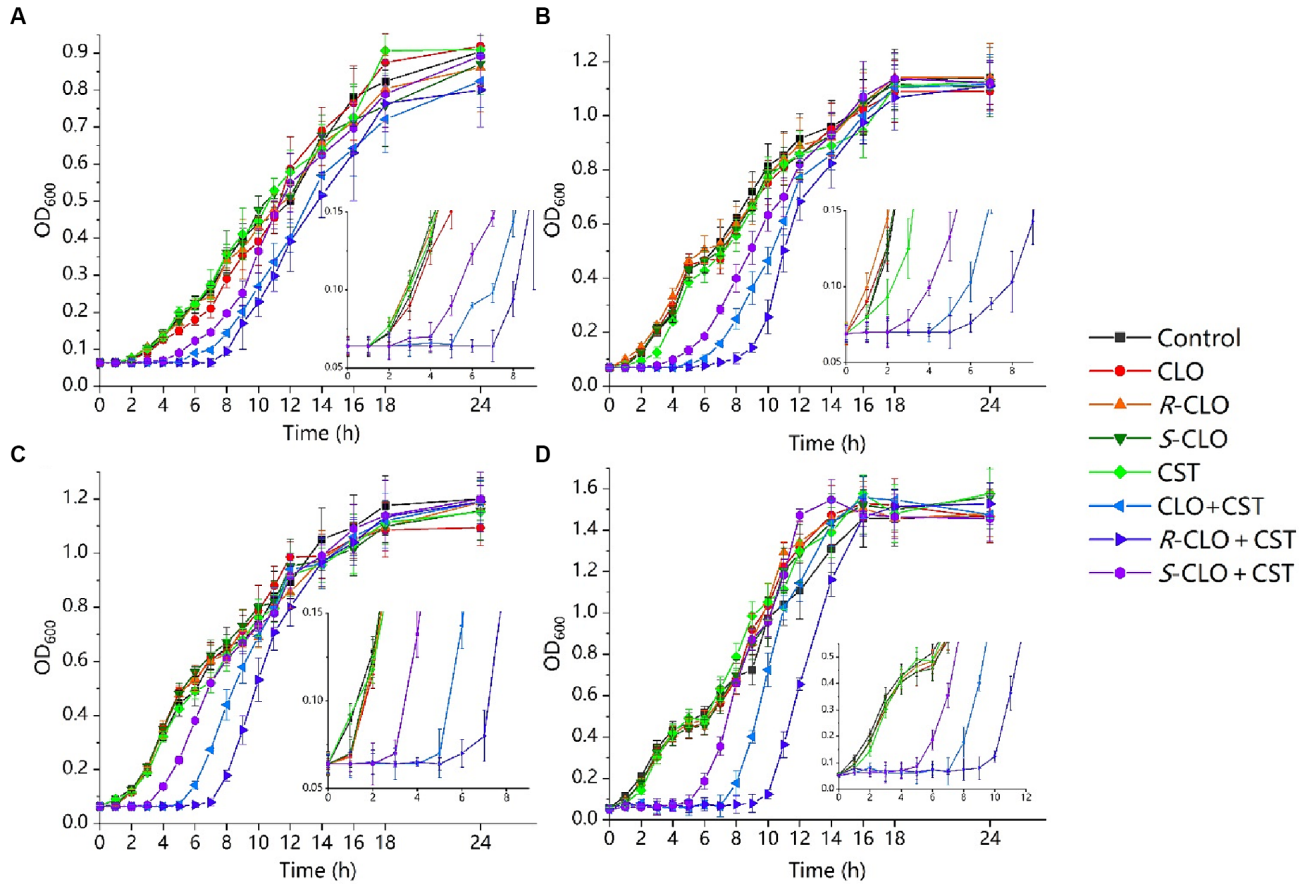
Growth inhibition assays for the single drug and combinations of CST with CLO, *R*-CLO, or *S*-CLO against four different GNB strains. **(A)** CST (0.1 μg/mL) with CLO (2 μg/mL) against *P. aeruginosa* PA02. **(B)** CST (0.1 μg/mL) with CLO (2 μg/mL) against *K. pneumoniae* K83. **(C)** CST (0.25 μg/mL) with CLO (2 μg/mL) against *E. coli* 44 (*mcr-1*). (d) CST (0.1 μg/mL) with CLO (2 μg/mL) against *E. coli* ATCC 25922. OD_600_ is the optical density at 600 nm. The drug concentration was determined through pre-experiments. CLO, closantel; *R*-CLO, *R*-closantel; *S*-CLO, *S*-closantel; CST, colistin.

To investigate the effect of CLO and its two enantiomers on bacterial growth, a growth inhibition assay was performed according to the protocol detailed in the methods section. According to the results of *in vitro* combined action three representative strains were selected from different genera and the standard strain *E. coli* ATCC 25922, describing the results in detail. As shown in Figure 3, 0.1 μg/mL CST, 0.25 μg/mL CST, and 2 μg/mL CLO, *R*-CLO, and *S*-CLO alone were unable to prevent bacterial growth. The addition of CLO and its two enantiomers substantially increased the growth inhibitory effect of CST on *P. aeruginosa* PA02, *K. pneumoniae* K83, *E. coli* 44 (*mcr-1*), and *E. coli* ATCC 25922. For all GNBs tested, the antibacterial activity of *R*-CLO + CST was higher than that of CLO + CST and *S*-CLO + CST.

#### 3.1.3 Time-killing curves of drug combinations against bacteria

**Figure 4:**
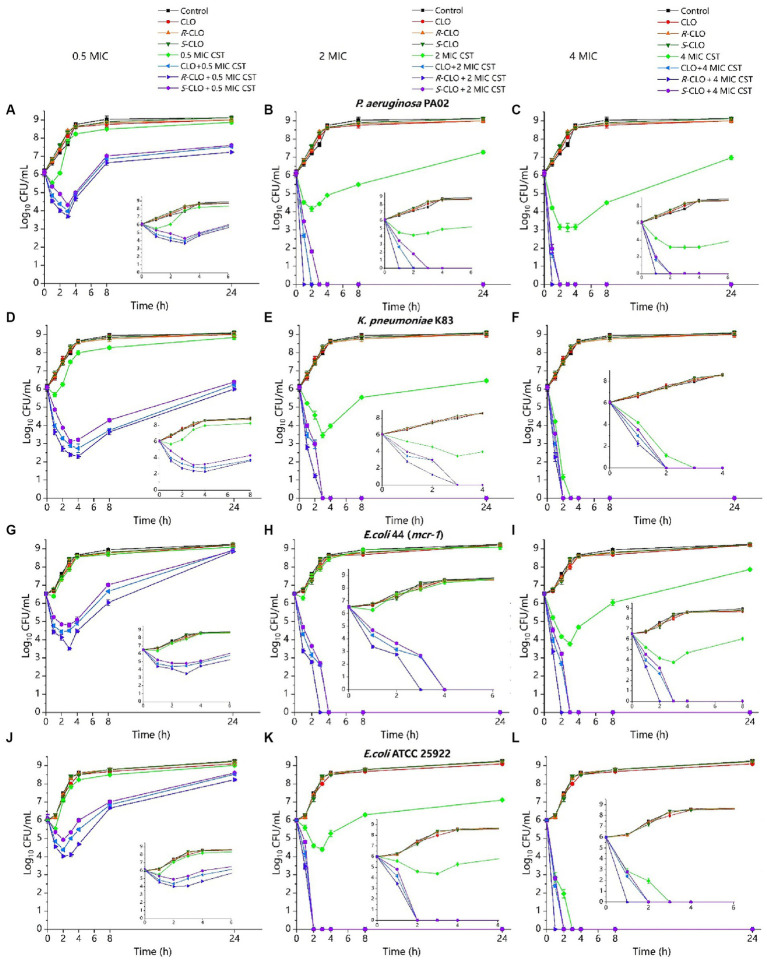
Time-killing curves of CST (0.5 MIC, 2 MIC, 4 MIC) and CLO (2 μg/mL) as single agents or in combination against bacteria. **(A–C)** *P. aeruginosa* PA02. **(D–F)** *K. pneumoniae* K83. **(G–I)** *E. coli* 44 (*mcr-1*). **(J–L)** *E. coli* ATCC 25922. Data are representative of three independent experiments and are shown as mean ± SD. CLO, closantel; *R*-CLO, *R*-closantel; *S*-CLO, *S*-closantel; CST, colistin.

We next explored the killing kinetics of CLO, *R*-CLO, *S*-CLO, and CST alone and in combination against GNB through time-kill curves. In all four strains, neither CST (0.5 MIC, 2 MIC) nor CLO and two enantiomers (2 μg/mL) alone were able to kill bacteria, whereas the introduction of CLO and its enantiomers at 2 μg/mL could effectively increase the bactericidal effect of CST against GNB (Figure 4).

For low concentrations of CST (0.5 MIC), compared with the single drug, the bactericidal effect of CST was increased by 3–4 orders of magnitude at the lowest cell density (Figures 4A,D,G,J). The difference between CLO, *R*-CLO, and *S*-CLO combined with 0.5 MIC CST was the most evident; the bactericidal effect of the *R*-CLO group was nearly an order of magnitude higher than that of the *S*-CLO group. The combination of high CST concentrations (2 MIC, 4 MIC) with CLO and its enantiomers yielded complete bacterial eradication within the first 3 h.

These checkerboards analysis, inhibition curves, and killing kinetics showed that the enantiomers of CLO were stereoselectivity antibacterial *in vitro* in combination with CST.

### 3.2 Exploration of mechanism

Mcr-1 gene is the first plasmid-mediated colistin resistance gene discovered in the world, which is a warning to the abuse of antibiotics. So, we chose *E. coli* 44 as a typical strain to explore the mechanism.

#### 3.2.1 Scanning electron microscopy analysis

**Figure 5:**
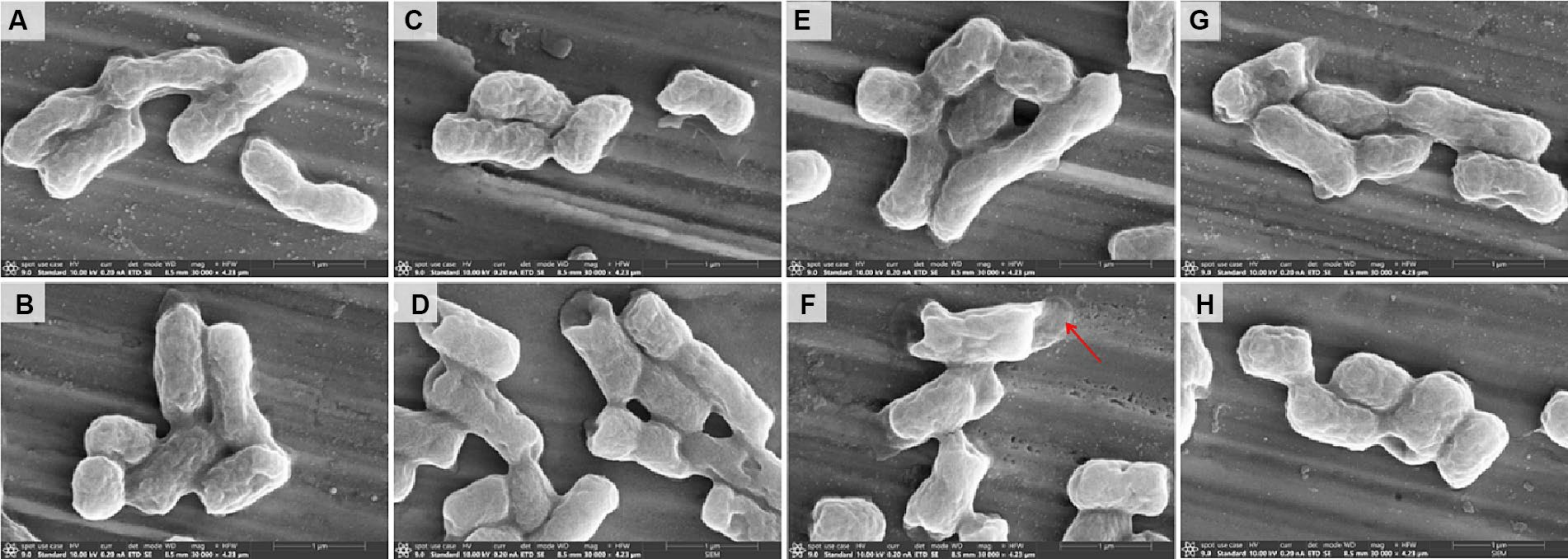
Morphological changes of **(A)** control and *E. coli* 44 (*mcr-1*) after sub-MIC of **(B)** CST, **(C)** CLO, **(D)** combination of CLO and CST, **(E)** *R*-CLO, **(F)** combination of *R*-CLO and CST, **(G)** *S*-CLO, **(H)** combination of *S*-CLO and CST treatment were visualized by SEM. Red arrows mark the outer membrane sites disrupted by combination of *R*-CLO and CST.

The morphological changes of the strain *E. coli* 44 (Figure 5) strains were analyzed by scanning electron microscopy (SEM) after treated with sub-MIC of CST, CLO, *R*-CLO, *S*-CLO or their combinations. The strain cells of the untreated control (Figure 5A) and the treated with CLO, *R*-CLO, *S*-CLO, and CST all remained intact, with complete cell membranes and plump cell morphology (Figures 5C,E,G). Compared with single-drug treatment, we observed that drug combination treatment had different degrees of damage to the outer membrane of cells (Figures 5D,F,H). For *E. coli* 44 strain, the combination of *R*-CLO and CST caused the most obvious damage to the bacterial outer membrane, while the combination of *S*-CLO and CST caused the least damage to the cells.

#### 3.2.2 ROS and ATP detection

**Figure 6:**
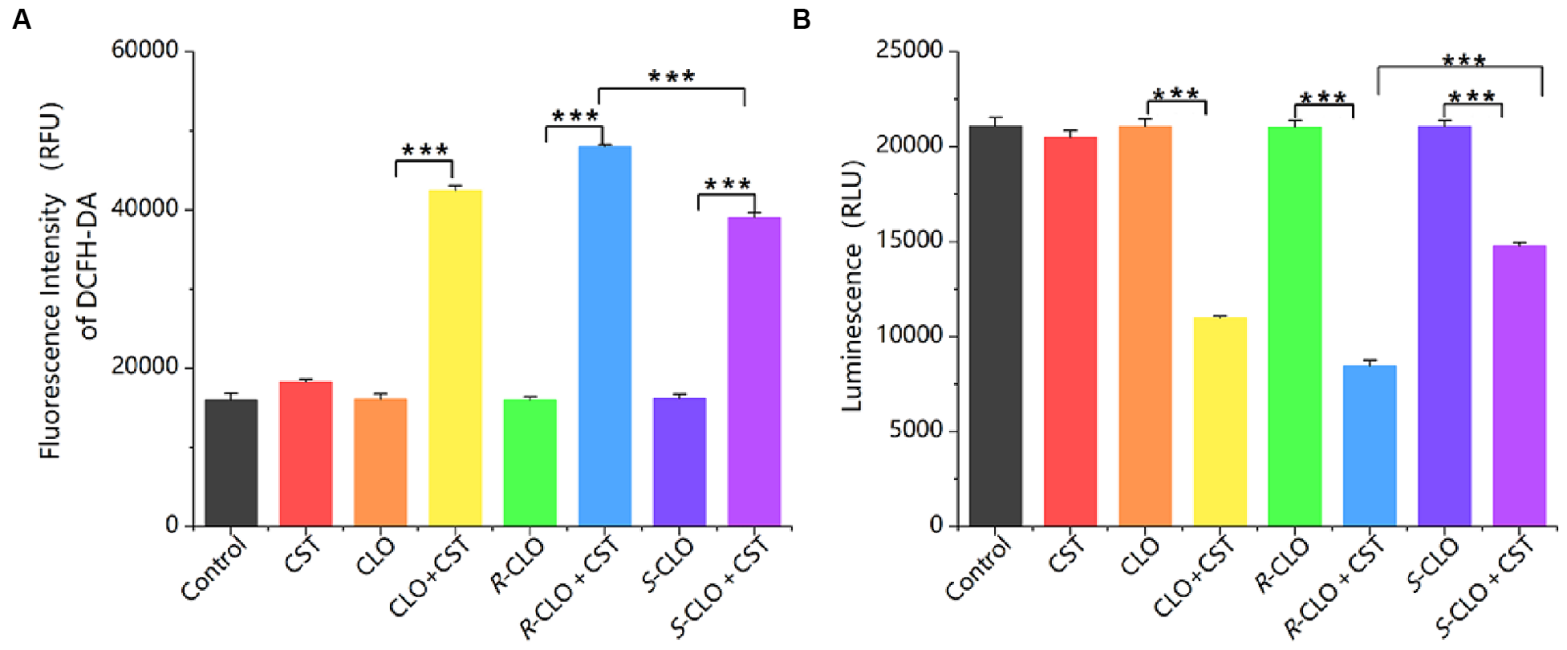
**(A)** ROS and **(B)** ATP production in *E. coli* 44 after treatment. One-way ANOVA was used to calculate *p*-values (***p < 0.001). CLO, closantel; *R*-CLO, *R*-closantel; *S*-CLO, *S*-closantel; CST, colistin.

ROS play a crucial role in the bactericidal action of antibiotics. Elevated ROS levels induce lipid peroxidation in cell membranes, leading to damage of critical biomolecules like proteins and DNA, ultimately causing bacterial cell death. DCFH-DA assays revealed that individual drug treatments did not significantly alter ROS levels. The findings indicate that the sole administration of drugs did not directly influence the overall ROS levels. In contrast, the combination of CLO, *R*-CLO, or *S*-CLO with CST significantly elevated total ROS production (Figure 6A). Furthermore, the fluorescence intensity of *R*-CLO + CST exceeded that of CLO + CST and *S*-CLO + CST (*p* < 0.001).

ATP is an essential substance for cell survival. A sharp decrease in intracellular ATP levels can lead to cell death, while a sharp increase can cause cellular damage through a series of compound reactions induced by the respiratory chain. The research findings indicate that the use of drugs alone does not directly affect the overall ATP levels in bacterial cells. In contrast, the combination of CLO, *R*-CLO, or *S*-CLO with CST significantly reduces intracellular ATP levels in bacterial cells and exhibits stereoselectivity (Figure 6B). *R*-CLO + CST results in significantly lower ATP synthesis within cells compared to CLO + CST and *S*-CLO + CST (*p* < 0.001).

#### 3.2.3 Detection of drug concentration in bacteria

**Figure 7:**
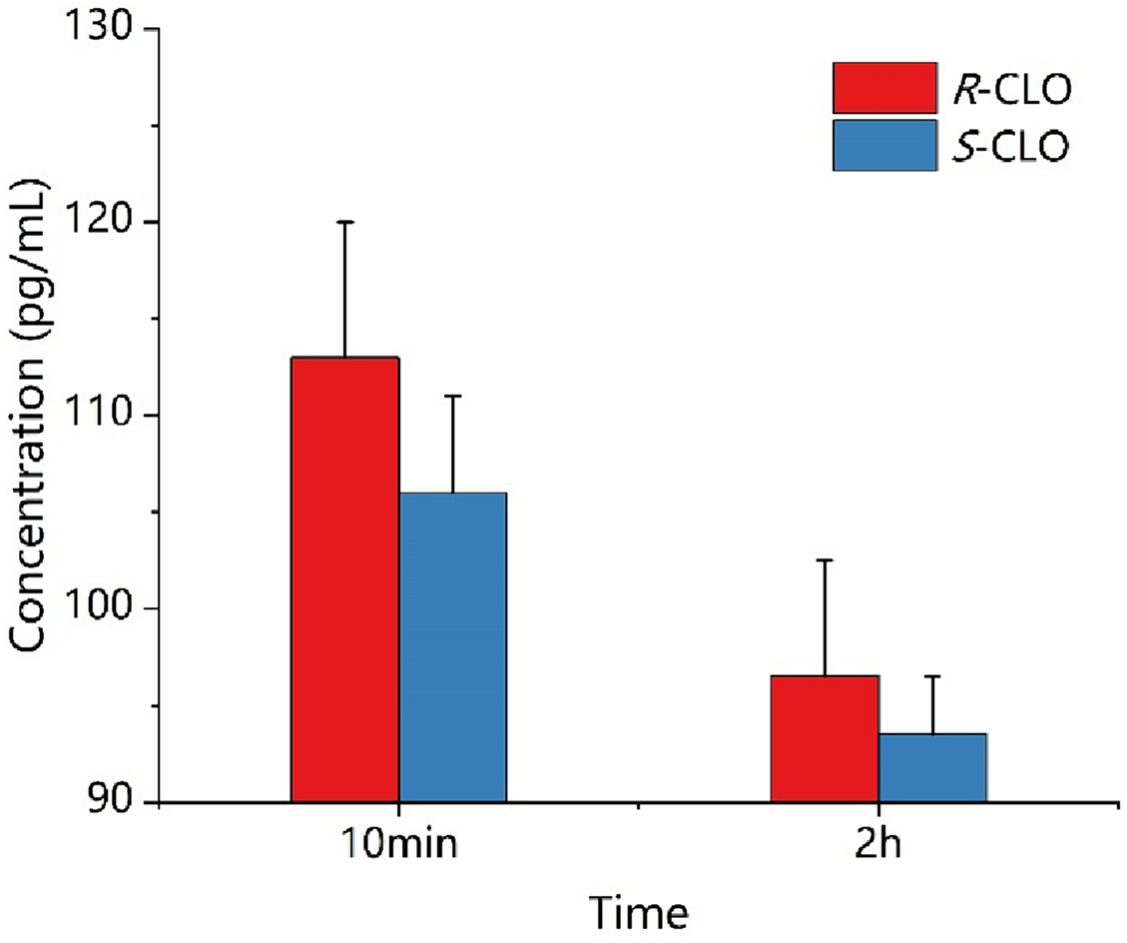
Concentration of enantiomer in *E. coli* 44 at different time. *R*-CLO, *R*-closantel; *S*-CLO, *S*-closantel.

As depicted in Figure 7, during a 10 min incubation of bacteria with the drug, the intracellular concentrations of *R*-CLO and *S*-CLO were 113 pg./mL and 106 pg./mL, respectively. After a 2 h incubation, the concentrations of *R*-CLO and *S*-CLO decreased to 96 pg./mL and 93 pg./mL, respectively.

### 3.3 *In vivo* pharmacodynamic studies

In the laboratory, we repeatedly modeled mouse leg abscesses using various strains. Successful modeling was achieved solely with the *P. aeruginosa* PA02 strain. This strain distinctly demonstrated the stereoselective capacity of CLO combined with CST in reversing drug resistance in GNB.

**Figure 8:**
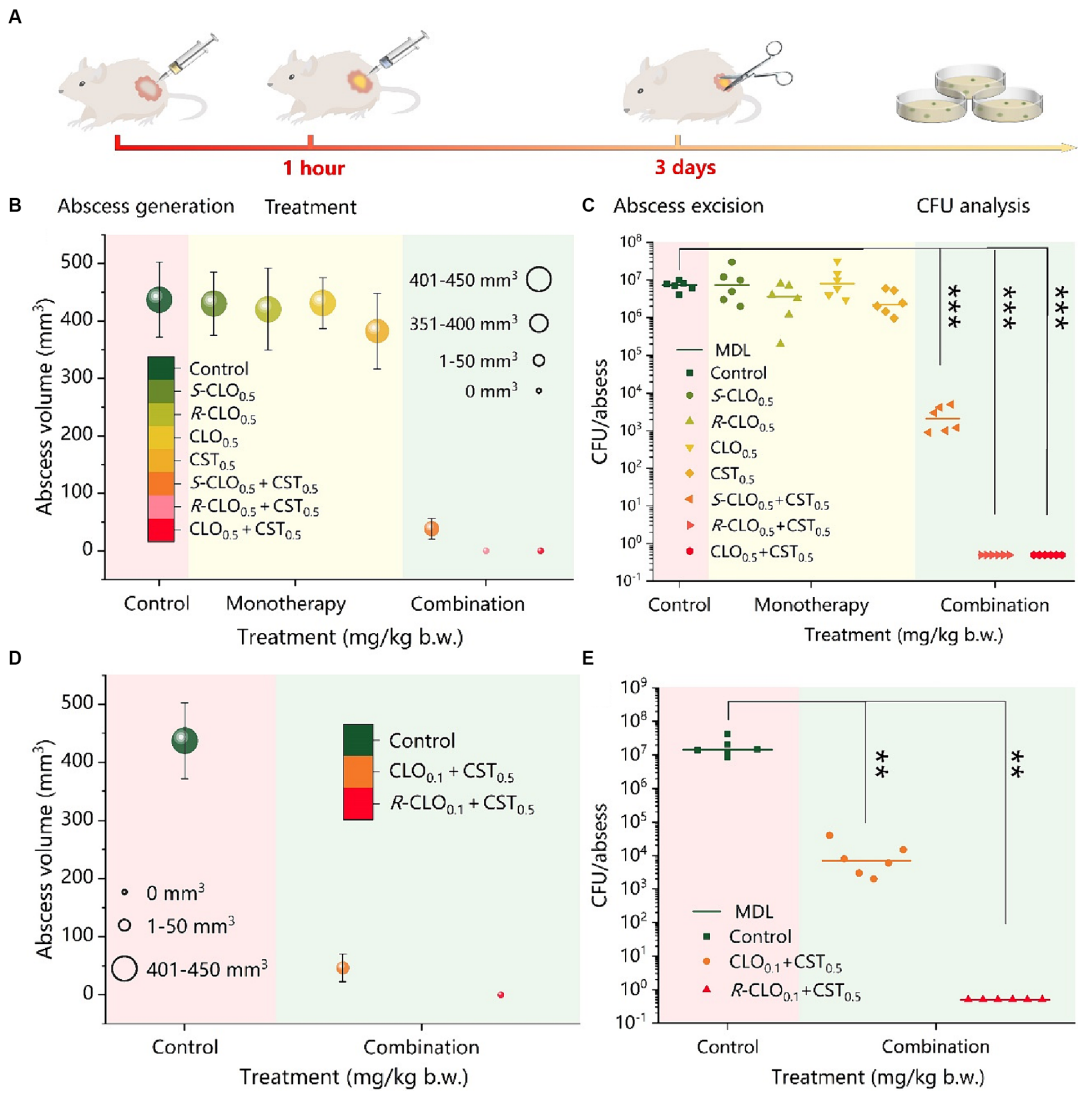
*In vivo* synergistic antimicrobial activity of CST and CLO, *R*-CLO, or *S*-CLO against CST-resistant *P. aeruginosa* PA02. **(A)** Schematic diagram of the construction of the mouse cutaneous infection model. **(B)** Abscess volume after monotherapy or combination therapy. **(C)** CFU per abscess in mice injected with CST (0.5 mg/kg b.w.) and 0.5 mg/kg b.w. CLO, *R*-CLO, or *S*-CLO alone or in combination, as indicated. The difference in the abscess volume **(D)** and CFU per abscess **(E)** were observed after the combination treatment of CLO or *R*-CLO with a dosage reduced to 0.1 mg/kg b.w. and 0.5 mg/kg b.w. of CST. Each experiment was conducted with six biological replicates, and the abscess volumes were the average of those from six mice. One-way ANOVA was used to calculate *p*-values (**p < 0.01, ***p < 0.001). CLO, closantel; *R*-CLO, *R*-closantel; *S*-CLO, *S*-closantel; CST, colistin.

A high-density mouse cutaneous infection model with CST-resistant *P. aeruginosa* PA02 was designed and established. The antibacterial synergy of the enantiomers with CST was demonstrated via subcutaneous injection in the murine model (Figure 8A). Both post-treatment abscess volume and colony-forming units (CFU) demonstrated significant differences in the synergistic antibacterial activity of the enantiomers and CST against *P. aeruginosa* PA02 *in vivo*, which was consistent with the *in vitro* results.

As shown in Figure 8B, the combination of CLO, *R*-CLO, or *S*-CLO with CST yielded significant synergistic antibacterial efficacy against the strain compared to that of monotherapy without a therapeutic effect. After CLO, *R*-CLO, and *S*-CLO monotherapy, the abscess volumes were 431, 420, and 430 mm^3^, respectively, which were very close to that of the control group (437 mm^3^). Furthermore, although the abscess volume was slightly reduced after CST treatment compared to that in the control group, it still exceeded 380 mm^3^. These observations suggest that these monotherapies are ineffective. After treatment with CLO_0.5_ + CST_0.5_ or *R*-CLO_0.5_ + CST_0.5_, no abscesses appeared in the skin lesions of mice (volume, 0 mm^3^). In contrast, the growth of abscesses was significantly inhibited after *S*-CLO_0.5_ + CST_0.5_ treatment, showing a volume of 38.3 mm^3^, implying that the abscesses were not fully healed. In addition, the abscess volume positively correlated with CFU. After treatment, the quantification of bacteria in the infected tissues of the control and monotherapy groups was comparable (approximately 10^7^ CFU), resulting in a larger abscess volume. The bacterial load in the *S*-CLO_0.5_ + CST_0.5_ treatment group was reduced by three orders of magnitude compared to that in the control group, reaching approximately 10^4^ CFU. This indicates that the combined drug limited the growth of abscesses by inhibiting bacterial proliferation (Figure 8C). Interestingly, the bacterial load of the mouse skin after CLO_0.5_ + CST_0.5_ or *R*-CLO_0.5_ + CST_0.5_ treatment was 0 CFU, implying that the abscess had healed. These observations highlight that CLO, *R*-CLO, and *S*-CLO have significant resistance-reversing effects *in vivo*. More importantly, the effect of *R*-CLO and *S*-CLO in reversing drug resistance differed, which was consistent with the *in vitro* results. The reversal of drug resistance by the enantiomers was stereoselective.

The abscess volume and CFU results, shown in Figures 8B,C, demonstrated that 0.5 mg/kg body weight (b.w.) CLO and *R*-CLO, in combination with CST, healed abscesses caused by *P. aeruginosa* PA02 (*p* < 0.001). Therefore, it is necessary to reduce the dosage of CLO and *R*-CLO to further explore the distinction in the antibacterial effect of the two in combination with CST. The combined *in vivo* antibacterial activity of 0.1 mg/kg b.w. CLO and *R*-CLO with 0.5 mg/kg b.w. CST was compared in the cutaneous infection model described above. As shown in Figures 8D,E, abscesses in the *R*-CLO_0.1_ + CST_0.5_ treatment group could still be cured (*p* < 0.01), and the corresponding bacterial burden was 0 CFU. However, for the CLO_0.1_ + CST_0.5_ group, the treated abscess was not completely eliminated, and the volume of the residual abscess was 46.5 mm^3^ with a bacterial burden of approximately 10^4^ CFU.

## 4 Discussion

Global public health security is severely threatened by the emergence and rapid spread of antibiotic resistance of GNB (Chen et al., 2023; Cai et al., 2023a,b). The repurposing of ‘old’ drugs as adjuvants that rescue antibiotics action MDR pathogens is a promising combination strategy for overcoming bacterial resistance (Wozniak et al., 2015; Tarín-Pelló et al., 2022). Domalaon et al. (2019) and Copp et al. (2020) studied the broad-spectrum anthelmintic drug CLO either alone or combined with CST against bacteria. They found that the combination of CST and CLO exerts a synergistic effect on a variety of CST-resistant GNB, even in *E. coli* strains carrying the *mcr-1* gene. The FICIs of the CST and CLO combination against *P. aeruginosa* PA02 and *E. coli* 44 (*mcr-1*) were 0.0097 and 0.1406, respectively. In this study, similar results were obtained when CLO (the racemate) was combined with CST against the typical GNB. To the best of our knowledge, this is the first study to show the stereoselectivity differences in the synergistic effects of the combination of enantiomers and colistin on different MDR resistant strains including *mcr-1*-positive strains.

*In vitro* growth inhibition and time-killing assays generally provide an initial and rapid indication of a drug’s bactericidal profile (Han et al., 2023). Consistent observations across all four tested GNB strains show that *R*-CLO enhances the antibacterial activity of CST more effectively than the CLO and *S*-CLO. SEM confirmed significant damage to the outer membrane of both standard and drug-resistant strains caused by combinations of CLO, *R*-CLO, and *S*-CLO with CST. This indicates that all enantiomers, in combination with CST, synergistically enhance the membrane-disruptive effects of CST. Notably, the fluorescence intensity of the *R*-CLO + CST combination surpassed those of CLO + CST and *S*-CLO + CST (*p* < 0.001), suggesting a more potent role of *R*-CLO in fostering oxidative damage and enhancing antibacterial efficacy. These results demonstrate that drug combinations have the potential to increase ROS levels in bacteria, leading to oxidative damage and heightened antibacterial efficacy. Furthermore, the fluorescence intensity of *R*-CLO + CST exceeded that of CLO + CST and *S*-CLO + CST (*p* < 0.001), signifying that, compared to CLO and *S*-CLO, *R*-CLO proves more effective in promoting oxidative damage to bacteria. This ultimately results in the superior antibacterial activity of the *R*-CLO + CST combination. This molecular mechanism is the primary way in which *R*-CLO and *S*-CLO exhibit stereoselectivity in reversing CST resistance.

The primary antimicrobial mechanism recognized for CST currently is: CST binds to the lipid A portion of LPS through electrostatic interactions. Subsequently, the fatty acyl tail chain of CST inserts into the phospholipid layer of LPS, disrupting the structure of the outer membrane lipid bilayer, thereby increasing membrane permeability. This leads to the leakage of bacterial cellular nutrients and physiological active substances, ultimately resulting in bacterial death (Stephani et al., 2023). On the other hand, CLO is an oxidative phosphorylation uncoupler that disrupts the electron transport chain of bacterial cells, promotes the dissipation of PMF, reduces ATP production, thus inhibiting bacterial growth, or directly killing bacteria (Levinson et al., 2019). For *E. coli* 44 (*mcr-1*) resistant to CST, the phosphate groups on lipid A of LPS are modified to phosphoethanolamine, reducing the negative charge on the surface of LPS. Positively charged CST finds it challenging to bind to LPS through electrostatic interaction, thereby disrupting the outer membrane structure. This has minimal impact on the stability and permeability of the outer membrane. Additionally, CLO finds it difficult to enter bacterial cells. Malla et al. (2020), through their research on reversing CST resistance using lipophilic proton carriers, proposed a hypothesis: CLO, similar to sodium salicylate, can facilitate the binding of CST to the outer membrane LPS target, thereby enhancing the antibacterial activity of CST. When *R*-CLO and *S*-CLO are used in combination with CST, under physiological pH conditions, both enantiomers carry negative charges. Due to their strong lipid solubility, they may first bind to the target site of LPS on the outer membrane of *E. coli*, which is resistant to CST, through hydrophobic interactions, thereby increasing the negative charge on the surface of LPS. This facilitates the electrostatic interaction between CST and the LPS target, thereby increasing the permeability of the outer membrane. Simultaneously, CST promotes the influx of CLO into the bacteria. The experimental results mentioned above indicate that the concentration of *R*-CLO and ROS inside the bacterial cell is higher than that of *S*-CLO, but the decrease in ATP levels is greater for *R*-CLO. Moreover, the damage to the surface morphology of bacterial cells is higher for *R*-CLO than *S*-CLO. These indicators suggest that *R*-CLO has a greater oxidative phosphorylation uncoupling intensity than *S*-CLO, thereby imparting stereoselective differences in synergistic antibacterial action between the two enantiomers of CLO and CST.

In combination with CST, *R*-CLO exerted the best therapeutic effect on abscesses, followed by CLO and *S*-CLO; however, both were significantly better than the monotherapy. The results demonstrate that the stereoselectivity of the synergistic antibacterial effect of enantiomers and CST is also observed *in vivo*. Notably, the combined antibacterial activity of the racemate was not the best, which highlights that the study of separate enantiomers regarding antibacterial effect is of great significance for providing more possibilities for its clinic use.

## 5 Conclusion

This study highlights the synergistic antibacterial effects of CLO and its enantiomers combined with CST against resistant GNB. *In vitro*, the combination significantly lowered MIC against strains like *E. coli* and *P. aeruginosa*, demonstrating enhanced bactericidal effects. *In vivo* tests using a mouse model echoed these findings, showing substantial reductions in abscess volumes and bacterial colonies. Particularly notable was the stereoselective efficacy of *R*-CLO compared to *S*-CLO in reversing drug resistance. These results underscore the potential of using chiral adjuvants to improve existing antibiotic therapies, especially against multi-drug resistant pathogens.

## Data availability statement

The original contributions presented in the study are included in the article/supplementary material, further inquiries can be directed to the corresponding authors.

## Ethics statement

The animal study was approved by South China Agricultural University Laboratory Animal Center (approval number: 2021c056). The study was conducted in accordance with the local legislation and institutional requirements.

## Author contributions

TD: Data curation, Formal analysis, Investigation, Methodology, Software, Validation, Writing – original draft, Writing – review & editing. ZG: Data curation, Formal analysis, Investigation, Methodology, Validation, Writing – original draft, Writing – review & editing. LF: Investigation, Supervision, Validation, Writing – review & editing. WG: Data curation, Validation, Writing – review & editing. YY: Writing – review & editing. YL: Conceptualization, Formal analysis, Methodology, Project administration, Resources, Validation, Writing – review & editing. XL: Writing – review & editing. LH: Conceptualization, Data curation, Formal analysis, Funding acquisition, Investigation, Methodology, Project administration, Resources, Software, Supervision, Validation, Visualization, Writing – review & editing.
